# Ocean circulation causes the largest freshening event for 120 years in eastern subpolar North Atlantic

**DOI:** 10.1038/s41467-020-14474-y

**Published:** 2020-01-29

**Authors:** N. Penny Holliday, Manfred Bersch, Barbara Berx, Léon Chafik, Stuart Cunningham, Cristian Florindo-López, Hjálmar Hátún, William Johns, Simon A. Josey, Karin Margretha H. Larsen, Sandrine Mulet, Marilena Oltmanns, Gilles Reverdin, Tom Rossby, Virginie Thierry, Hedinn Valdimarsson, Igor Yashayaev

**Affiliations:** 1 National Oceanography Centre, Southampton, UK; 2 Institute of Oceanography, University of Hamburg, Hamburg, Germany; 3 Marine Scotland Science, Aberdeen, Scotland UK; 4 Department of Meteorology and Bolin Centre for Climate Research, Stockholm University, Stockholm, Sweden; 5 Scottish Association for Marine Science, Oban, Scotland UK; 6 Faroe Marine Research Institute, Tórshavn, Faroe Islands; 7 Department of Ocean Sciences, Rosenstiel School of Marine and Atmospheric Science, University of Miami, Coral Gables, FL USA; 8 CLS, Ramonville-Saint-Agne, France; 9 Ocean Circulation and Climate Dynamics, GEOMAR Helmholtz Centre for Ocean Research Kiel, Kiel, Germany; 10 Sorbonne Université, CNRS/IRD/MNHN (LOCEAN), Paris, France; 11 Graduate School of Oceanography, University of Rhode Island, Kingston, USA; 12 Ifremer, Univ. Brest, CNRS, IRD, Laboratoire d’Océanographie Physique et Spatiale, IUEM, France; 13 Marine and Freshwater Research Institute, Reykjavik, Iceland; 14 Bedford Institute of Oceanography, Dartmouth, NS Canada

**Keywords:** Climate sciences, Ocean sciences, Physical oceanography

## Abstract

The Atlantic Ocean overturning circulation is important to the climate system because it carries heat and carbon northward, and from the surface to the deep ocean. The high salinity of the subpolar North Atlantic is a prerequisite for overturning circulation, and strong freshening could herald a slowdown. We show that the eastern subpolar North Atlantic underwent extreme freshening during 2012 to 2016, with a magnitude never seen before in 120 years of measurements. The cause was unusual winter wind patterns driving major changes in ocean circulation, including slowing of the North Atlantic Current and diversion of Arctic freshwater from the western boundary into the eastern basins. We find that wind-driven routing of Arctic-origin freshwater intimately links conditions on the North West Atlantic shelf and slope region with the eastern subpolar basins. This reveals the importance of atmospheric forcing of intra-basin circulation in determining the salinity of the subpolar North Atlantic.

## Abstract (web summary)

The Atlantic Ocean overturning circulation is important to the global climate system. Here the authors show that eastern subpolar North Atlantic underwent extreme freshening during 2012 to 2016, with a magnitude never seen before in 120 years of surface measurements.

## Introduction

The high salinity of the northward-flowing upper waters of the North Atlantic ocean is an essential condition for the formation of deep, cold, dense waters at high latitudes, as part of the meridional overturning circulation (MOC)^1,2^. Models have shown that the addition of freshwater to the upper layer of the subpolar North Atlantic (SPNA, 47–65°N, 0–60°W) could reduce the salinity sufficiently that atmospheric cooling results only in a cold, fresh, light upper layer. In turn this potentially weakens deep convection in the Nordic Seas (65–80°N, 25°W–20°E) and the SPNA, and the density of the deep western boundary currents, leading to a reduction in the MOC and the associated heat transport^3–5^.

During 2012–2016, the upper 1000 m of the SPNA acquired an extra 6600 km^3^ of freshwater; a rate of change and volume at a magnitude that has not been observed since the late 1960s. The distribution of the additional freshwater in 2012–2016 is not uniform over the SPNA, and tracing the development of the signal and its propagation during the modern period of a well-observed ocean allows us to identify the mechanisms that led to its development.

The North Atlantic upper ocean acquires its high salinity signature through a combination of the advection of saline water from the Indian Ocean^6^, MOC processes in the South Atlantic^2^, and the removal of freshwater from the surface in the subtropics by evaporation and subsequent atmospheric transport to the Pacific^2^. Over long timescales, the addition of salt and removal of freshwater is approximately balanced by the introduction of freshwater from the Arctic Ocean via the shallow Labrador Current (LC) and East Greenland Current (including ice sheet melt water), the import of freshwater from the Southern Ocean by the South Atlantic subtropical gyre, and SPNA net precipitation.

The North Atlantic Current (NAC^7^) forms the southern and eastern boundary current of the subpolar gyre circulation. The NAC formation zone lies northeast of the Grand Banks and Flemish Cap, where the subtropical gyre western boundary current (the Gulf Stream) turns sharply east. The NAC widens as it crosses the North Atlantic, separating into branches east of the Mid-Atlantic Ridge that flow into the Iceland Basin, the Rockall Trough and southward to re-join the subtropical gyre^8^.

The processes that supply salt and freshwater to the SPNA are subject to temporal variations, and the net effect is the salinity change that has been observed over interannual to decadal timescales^9–14^. The SPNA underwent a freshening period from the late 1960s to the mid-1990s^14,15^, followed by a decade of increasing salinity^16^. The rapid upper layer freshening of the late 1960s was termed the Great Salinity Anomaly, and has been associated with a reduction in Labrador Sea winter convection in subsequent years^3,17^. That event, along with the following prolonged period of low SPNA salinity was shown to originate from increased precipitation and river runoff in the Arctic that was subsequently exported to the south^18^. Interannual variability is superimposed on decadal changes, with notable minima in salinity in the 1980s and 1990s^19^.

There is a hypothesised link between varying freshwater export from the Arctic and SPNA salinity^18^. Increasing salinity (reduced freshwater content) in the SPNA from mid-1990s to late 2000s coincided with the accumulation of freshwater in the Arctic Ocean^20,21^. At the same time, the transport of fresh Arctic water into the SPNA via the Canadian Arctic Archipelago and LC was low compared to the long-term (70 year) mean transport^22^.

The large-scale ocean circulation has been invoked to explain long term North Atlantic centennial to decadal property changes^23^; a weaker MOC is associated with decreased ocean heat transport convergence and reduced SPNA heat storage and basin-wide sea surface temperature^24–26^. There is evidence that periods of low SPNA salinity in the twentieth century arose in part from a reduction in northward transport of salt from the subtropics by the MOC, associated with changes in wind forcing^27^. Even with a constant import of Arctic water, when the MOC slows down less fresh water is transported southwards in the deeper layers and so imported Arctic water is retained in the SPNA gyre^28^. A recent analysis argues that a reduced MOC at 26°N after 2008 led to subsequent cooling and freshening of the eastern SPNA^29^. However, during 2014–2016 the reported convergence of ocean freshwater transport between two MOC observational arrays (RAPID at 26°N^29^ and OSNAP at 53–60°N^30^) appears to be too low to account for a large change in SPNA freshwater storage.

The interaction with the atmosphere is also important; changes in net precipitation contribute to salinity changes^31^. The North Atlantic Oscillation^32^ (NAO), the first leading mode of atmospheric variability, is a key driver of change and its associated patterns of local wind stress, heat loss and net precipitation can have a cumulative effect over several years^33^. The East Atlantic Pattern^34^, the second leading mode of atmospheric variability, is thought to regulate the subpolar gyre circulation and the leakage of subtropical waters into the SPNA^11^.

Within the SPNA region, atmospheric forcing (particularly the NAO and the associated wind stress curl) can alter the regional distribution of salinity through changes in the zonal spread of water masses and shifts in the location of the NAC in the Newfoundland Basin and the Iceland Basin/Rockall Trough region^10,33–37^. The NAC forms a boundary zone (Subpolar Front) between the Arctic-influenced cool, fresher waters of the western and central SPNA and the subtropical-influenced warm, saline waters.

Despite the conceptual link between the salinity of the SPNA and the transport of freshwater from the Arctic and salt from the subtropics, we lack detailed knowledge of the processes and how they change over time. Here we examine the mechanisms that determine the temporal variability of the SPNA upper ocean salinity (0–1000 m) through an extraordinarily strong freshening event observed from 2012 to 2016; the fastest and greatest change in the salinity of the eastern SPNA in 120 years. We show that the cause of the change was unusual winter wind patterns driving major changes in ocean circulation.

## Results

### Freshening of the eastern subpolar region in 2012–2016

**Fig. 1:**
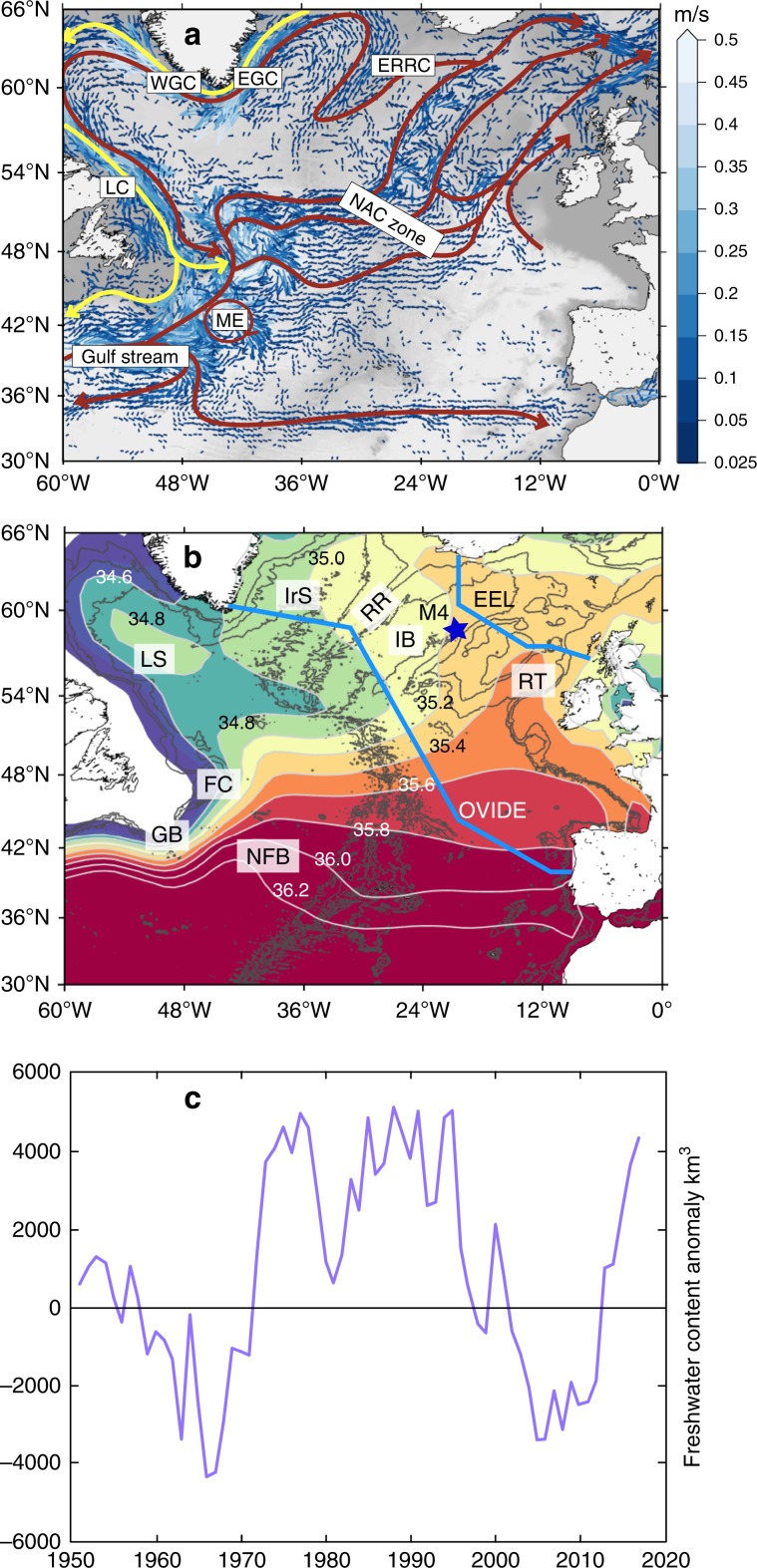
North Atlantic circulation, surface salinity and changing freshwater content. **a** mean circulation of the North Atlantic Ocean illustrated with mean surface current vectors overlain by schematic representation of major currents including the North Atlantic Current (NAC), East Reykjanes Ridge Current (ERRC) East Greenland Current (EGC), West Greenland Current (WGC), Labrador Current (LC) and Mann Eddy (ME); **b** mean surface salinity field from the EN4 dataset (2004–2016) with major bathymetric features labelled, including Labrador Sea (LS), Irminger Sea (IrS), Reykjanes Ridge (RR), Iceland Basin (IB), Rockall Trough (RT), Flemish Cap (FC), Grand Banks (GB) and Newfoundland Basin (NFB), the Extended Ellett Line (EEL) and OVIDE sections (blue lines), and the M4 mooring (blue star); **c** annual time series of subpolar North Atlantic freshwater content of the upper 1000 m, derived from the EN4 dataset and integrated over the region 47–65°N, 0–60°W (see Methods). Figure 2, annual salinity anomalies in the 0–200 m layer. Data from the EN4 dataset, mean period (shown top left) is 2005–2016.

**Fig. 2:**
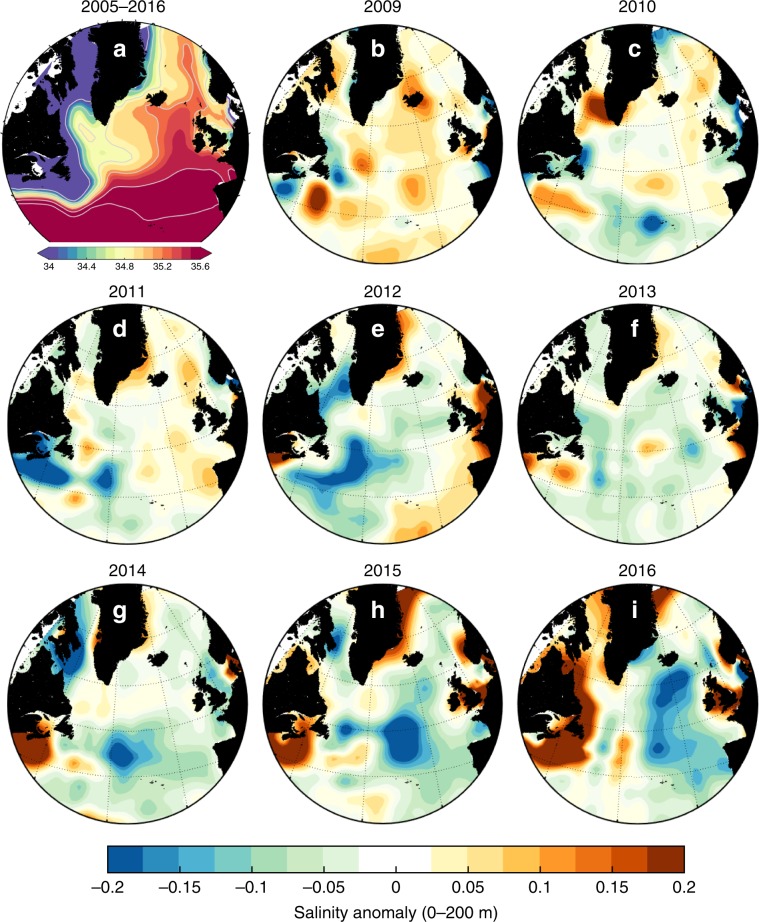
The mean salinity of the shallow upper ocean in the subpolar North Atlantic, and the evolution of anomalies between 2009 and 2016. **a** mean salinity in the 0–200 m depth zone for the period of 2005–2016, computed from the EN4 dataset. **b**–**i** salinity anomalies of the 0–200 m layer in each year from 2009 to 2016, referenced to the 2005–2016 mean; warm colours represent higher salinity and cool colours are lower salinity. In 2012 a strong negative salinity anomaly developed in the Newfoundland Basin, and by 2016 it had propagated to the Iceland Basin and Rockall Trough. A strong positive anomaly developed in the North West Atlantic continental shelf and slope region during 2014–2016.

**Fig. 3:**
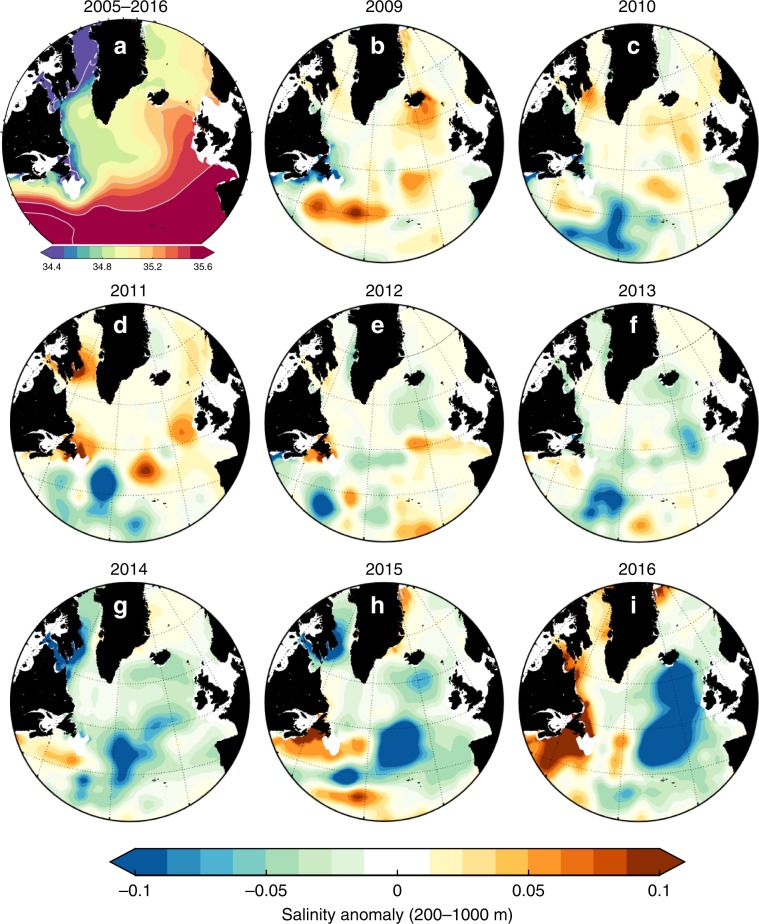
The mean salinity of the 200–1000 m depth zone of the subpolar North Atlantic, and the evolution of anomalies between 2009 and 2016. **a** mean salinity in the 200–1000 m zone for the period of 2005 to 2016, computed from the EN4 dataset. **b**–**i** salinity anomalies of the 200–1000 m zone in each year from 2009 to 2016, referenced to the 2005–2016 mean; warm colours represent higher salinity and cool colours are lower salinity. Note that the colour scale here is different to that shown in Fig. 2. A strong negative salinity anomaly developed in the Newfoundland Basin in 2014 (2 years later than the 0–200 m negative anomaly), and by 2016 it had propagated to the Iceland Basin and Rockall Trough. A positive anomaly developed in the North West Atlantic continental shelf and slope region during 2014–2016.

Integrated across the SPNA, the freshwater content of the upper 1000 m increased rapidly by ~6600 km^3^ during 2012–2016 (Fig. 1). However, the change in salinity was not distributed evenly over the region. The evolution of the salinity field in two layers, 0–200 m and 200–1000 m (anomalies from a long-term mean derived from the EN4 data sets, see Methods) is shown in Figs. 2 and 3. During 2005–2009, the SPNA was characterised by elevated salinity and low freshwater content in the upper 1000 m (Fig. 1). In 2009–2010, the North West Atlantic Continental Shelf and Slope region (NWACSS, 40–50°N, 50–70°W, which includes offshore deep water) was relatively fresh, and the basins of the central subpolar gyre (the Labrador and Irminger Seas) had salinities close to the long-term mean (Figs. 2, and 3). In 2011, a fresh anomaly formed in the 0–200 m layer of the Gulf Stream and between 40 and 45°N in the Newfoundland Basin in both layers. In 2012, the 0–200 m layer of the Newfoundland Basin was observed to be very fresh. Notably in 2013 the NWACSS region switched to saline conditions while most other regions were slightly fresher than the long-term mean. The freshening in the Newfoundland Basin intensified in 2014–2016 and extended to the Mid-Atlantic Ridge region (20–40°W, 40–50°N) in both layers. The fresh feature propagated along the NAC pathway reaching the Iceland and Rockall Basins in 2015 and intensifying in both layers in 2016. In contrast, the central subpolar gyre (the Labrador Sea and Irminger Sea) was largely characterised by salinity close to the decadal (2005–2016) mean, while salinities off the coast of Labrador increased.

The EN4 profile data sets inclusive of all available Argo float data provide good annual information over a wide region. However, to resolve the location and timing of subsurface salinity anomalies associated with this freshening event with greater confidence, we turn to high resolution, high quality observations at fixed locations and transects.

**Fig. 4:**
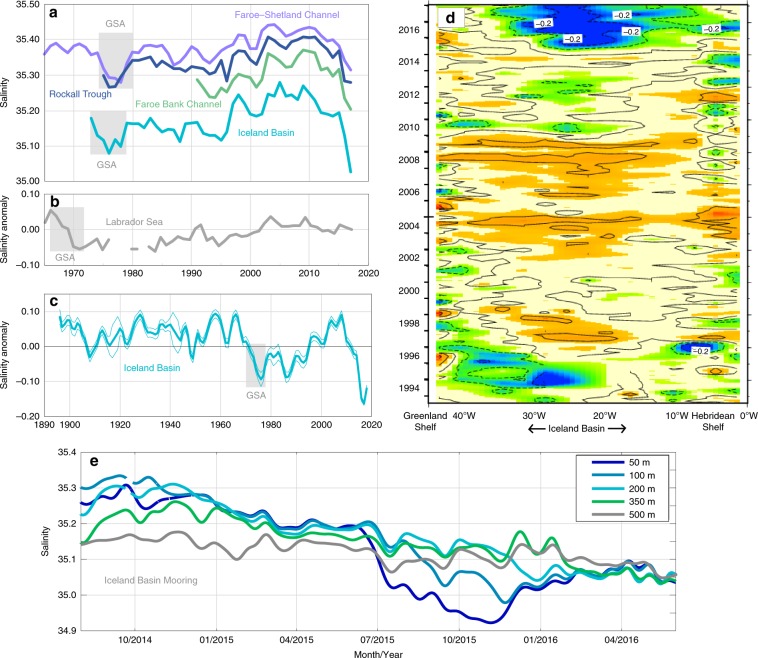
Time series records of North Atlantic salinity. **a** annual mean upper ocean salinity from repeat hydrography sections in the Faroe Shetland Channel (North Atlantic Water salinity core, 0–200 m at 61°N 3°W), Rockall Trough (30–800 m at 57.5°N 11.0°W), Faroe Bank Channel (North Atlantic Water salinity core at 61.4°N 8.3°W) and Iceland Basin (Station Selvogsbanki 5, 63.0°N 21.5°W, 0–200 m), grey boxes indicate the Great Salinity Anomaly; **b** annual upper ocean (0-500 m) salinity anomaly (from seasonal means) from the interior Labrador Sea; **c** annual sea surface salinity records from the Iceland Basin (irregular polygon approximately 52-64°N, 10–20°W, thin lines indicate measurement error estimate); **d** Time-longitude plot of sea surface salinity anomaly at 60°N between Greenland and the Hebridean Shelf (mean is computed over 1993-2017, contour interval 0.1 psu, solid black line is zero). **e** A 2-year continuous record (2014–2016) of salinity observations at depths between 50 and 500 m at the M4 OSNAP mooring in the Iceland Basin (58.0°N, 21.1°W).

First we consider the magnitude of this freshening event in a multidecadal context. Time series from the very small number of sections and stations with more than four decades of high quality subsurface observations, show that the 2012–2016 freshening event is the largest and most rapid change in salinity (up to −0.25) observed for 45 years (Fig. 4a), i.e. since the Great Salinity Anomaly that started in the late 1960s and progressed through the SPNA during the 1970s (also visible as a rapid increase in freshwater content, Fig. 1c). All the time series in Fig. 4a show a period of increasing salinity during the 1990s and 2000s, and decadal-scale freshening after 2008. However, unlike the Great Salinity Anomaly, the recent accelerated freshening signal is restricted to the eastern basins (Iceland Basin, Rockall Trough) and downstream into the southern Norwegian Sea (Fig. 2); there is no similarly large signal in the Labrador Sea as of 2016 (Fig. 4b). Multidecadal records of surface salinity from the Iceland Basin indicated that this is actually the lowest salinity in 120 years of observations, and thus is a highly unusual event in this time frame (Fig. 4c). A 25-year record of surface salinity changes on a ship-of-opportunity route at 60°N between Greenland and north of Scotland (Fig. 4d) illustrates not only the extraordinary magnitude of the surface salinity anomaly (up to −0.2), and its regional focus in the eastern basins of the SPNA, but also its rapid onset in 2015 in the Iceland Basin. There is no evidence of any change in surface salinity in the East Greenland Current (Figs. 1a and 4d) or on the Hebridean shelf, which are located outside of the NAC zone.

The time frame in which the salinity anomaly arrived in the Iceland Basin is further illustrated by mooring records from OSNAP (Overturning in the Subpolar North Atlantic Programme)^29^. The freshening event evolved most rapidly in the upper 200 m layer where the salinity decreased from 35.30 when the moorings were deployed in summer 2014, to just 35.05 2 years later (Fig. 4e). Part of that reduction in salinity (nearly 0.2), took place in an extremely high magnitude, fast event between July and November 2015. The fresh water advection was concentrated near the surface during the stratified 2015 summer season, and then mixed deeper in the winter (temporarily causing a rise in the surface salinity in December 2015).

**Fig. 5:**
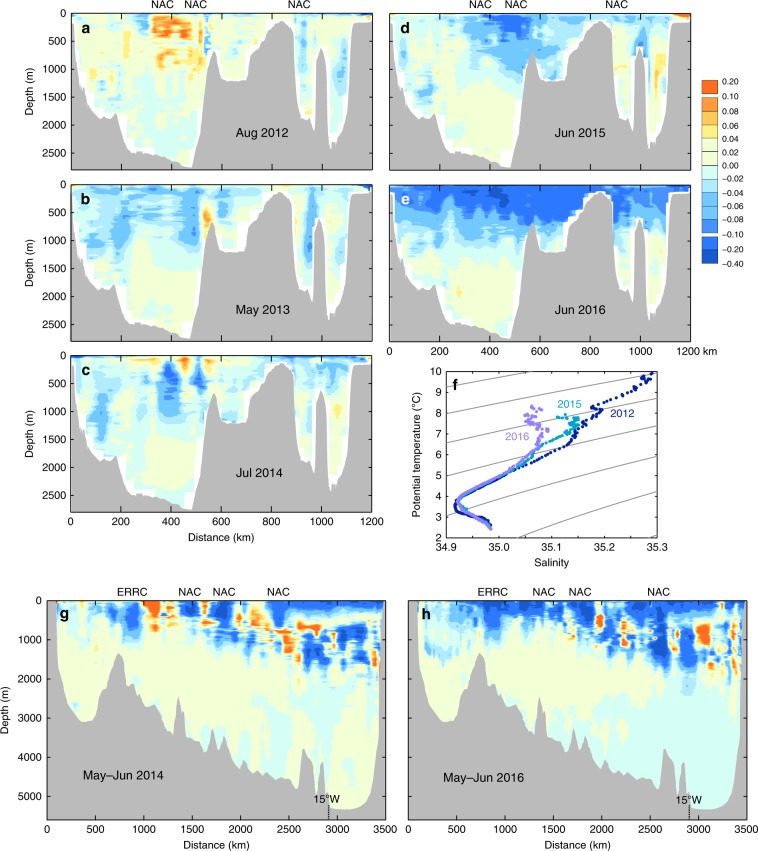
Detailed spatial structure of the salinity anomalies. **a**–**e** salinity anomalies at the Extended Ellett Line (EEL) annually repeated hydrography section (mean period is 2004–2017); (f) Potential temperature vs. salinity for EEL sections in 2012, 2015 and 2016 in the east Iceland Basin (located at 400–550 km, excluding seasonally-warmed upper 50 m); **g**–**h** salinity anomalies at the OVIDE section in May–June 2014 and May–June 2016 (mean period is 2002–2012, colour scale is same as **a**–**e**). Location of North Atlantic Current (NAC) and East Reykjanes Ridge Current (ERRC) branches closely tied to topography are indicated. Locations of the sections are shown in Fig. 1b.

Next we examine how salinity anomalies relate to the circulation features of the SPNA; are they evenly spread across the broad NAC zone (as suggested by EN4) or constrained in location by mode waters, eddies or density fronts? Ship-based summer hydrographic sections provide high basin-wide spatial resolution of the anomalies. Data from the OVIDE^8^ and Extended Ellett Line^12^ sections (Fig. 5a-e) show that the entire 0–1000 m zone to the east of the Mid Atlantic Ridge (including the Iceland Basin and Rockall Trough) is −0.20 to −0.30 fresher than climatology by 2016. These high-resolution data show that anomalies first appear in 2014 constrained to the location of the NAC jets in the east and mid Iceland Basin, in the southward flowing East Reykjanes Ridge Current, and at the southern end of the OVIDE section (Fig. 5g, h) where the magnitude of the anomaly in the upper 200 m (−0.08 in OVIDE 2014 and up to −0.1 in OVIDE 2016, Fig. 5g, h) matches the EN4 data very well (−0.075 in 2014 and −0.1 in 2016, Fig. 2). The type of water present in the east Iceland Basin NAC branch changed rapidly to a colder, fresher variety, as indicated by the temperature-salinity relationship (Fig. 5f).

**Fig. 6:**
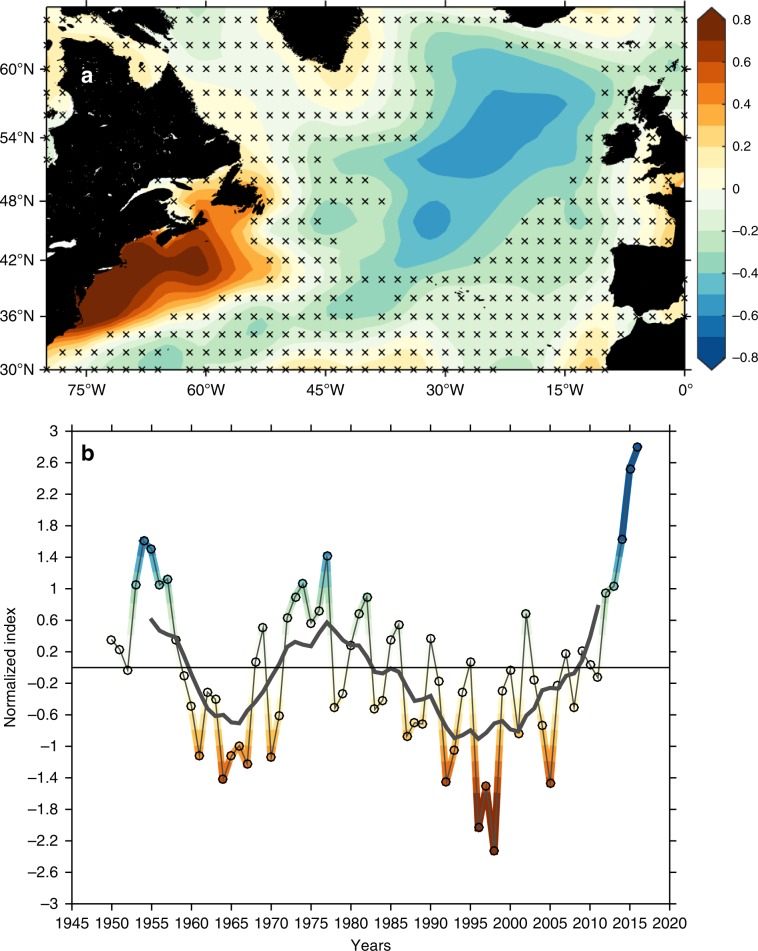
The leading empirical orthogonal function mode based on EN4 0-200 m annual mean salinity in the North Atlantic since 1950. This leading mode explains 31.7% of the variance. **a** Correlation analysis between the first principal component and the 0–200 m salinity field. Crosses indicate insignificance. **b** Salinity first principal component (coloured) overlaid by the 11-year running mean (thick grey). Positive phases (blue colours) project onto a dipole pattern with fresher eastern subpolar North Atlantic basins and more saline North West Atlantic Continental Shelf and Slope region, and vice versa.

A Principal Component Analysis of the North Atlantic salinity field since 1950 shows a spatial pattern for the leading mode of variability with centres of action in the Iceland Basin and the NWACSS (Fig. 6). The time series of the EOF, and the multidecadal records in the Iceland Basin (Figs. 4 and 6) confirms that the 2012–2016 event far exceeds the scale of any freshening in the past 70–120 years. The relationship between decadal-scale changes in salinity in the Iceland Basin and the NWACSS points towards processes, which have not been previously recognised or identified. In the next section, we identify the mechanisms that caused the recent acceleration of eastern SPNA freshening.

### Formation mechanisms of the 2012–2016 salinity anomaly

The fate of the Arctic-origin freshwater in the LC (hereafter referred to as LC-Arctic) is to either leave the shelf break and enter the subpolar circulation or to continue tracing the shelf-edge, interacting with saline Gulf Stream water in the NWACSS region^38,39^. On the Newfoundland and Labrador shelf, the LC-Arctic flows along the shelf break as a surface-intensified, fresh and buoyant baroclinic current in the upper 300 m, adjacent to an offshore (more saline) barotropic current which forms part of the boundary circulation of the Labrador Sea and subpolar gyre^22,40^. Some part of the LC-Arctic is diverted offshore and into the open ocean between the Flemish Cap and south of the Grand Banks^38,41^. Offshore of those topographic features, in the Newfoundland Basin, is the location where a negative salinity anomaly began to rapidly develop in 2012 (Fig. 2). Notably the anomaly was initially restricted to the depth zone matching the surface-intensified LC-Arctic (0–200 m). At the same time the NWACSS to the south of the SPNA became anomalously saline, indicating that this region was likely receiving less freshwater from the LC-Arctic than in previous years (and less oxygen^41^).

This implies an unusual diversion of Arctic freshwater in the LC away from the NWACSS and into the interior SPNA during 2012–2016. The NWACSS region freshwater content anomaly was −4600 km^3^ in 2012–2016, while the eastern SPNA region anomaly was +5900 km^3^, i.e. the re-routing of the LC-Arctic water was a major contributor to the increase in the total SPNA freshwater content anomaly and the accelerated freshening in the eastern basins. The LC freshwater that was diverted offshore joined the NAC at the North West Corner and was advected eastward, as indicated by the strong negative salinity anomalies arriving within the eastern NAC jets in 2014 and 2015 (Fig. 5). This mechanism of changing the pathway of Arctic water in the LC, such that the NWACSS received less Arctic freshwater inducing an increase in salinity, explains the dominant spatial salinity pattern (dipole-like between NWACSS and Iceland Basin) of long-term changes in the North Atlantic (Fig. 6).

**Fig. 7:**
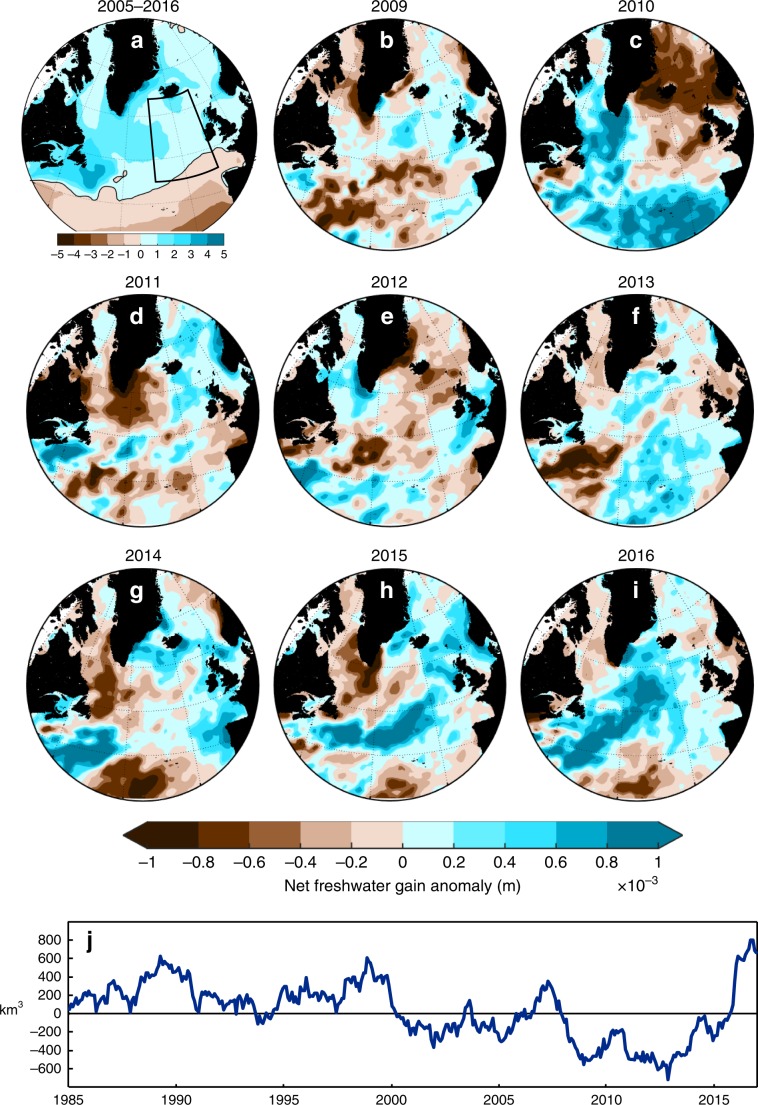
Freshwater input from the atmosphere. **a** 2005–2016 mean net freshwater gain by the ocean (precipitation minus evaporation) from ERA-Interim dataset. **b**–**i** 2009–2016 annual anomalies referenced to the mean **j** a 5-year moving sum of monthly net precipitation anomalies (from 1981-2010 mean) integrated over the region 45–65°N, 30–10°W (box outlined in **a**).

Next we consider changes in net precipitation. The SPNA has a climatological net gain of freshwater (higher precipitation than evaporation^42^). There are large interannual and patchy changes in net precipitation 2009–2014 (Fig. 7). In 2015 and 2016 it is noteworthy that the pathway of the NAC zone received particularly high levels of excess freshwater. In the eastern basins there was a freshwater gain anomaly totalling 700 km^3^ during 2012–2016, representing 10% of the freshwater content change (0–1000 m) over the whole SPNA. We conclude that an increase in net precipitation is a minor mechanism for increased freshwater content, but one that reinforced the signal driven by circulation changes (Fig. 7).

A major source of freshwater to the upper 1 km of the SPNA is the Arctic water that enters the region in the shallow, buoyant East Greenland Current and LC (Fig. 1a). A change in the amount of freshwater entering the SPNA could lead to a freshwater content anomaly. We examine whether Arctic sources are a major component of the change in SPNA freshwater content by looking for evidence of changes in freshwater transport within those currents, and for evidence of propagating salinity anomalies where those waters circulate.

There were no clear changes in the transport of Arctic freshwater into the SPNA through the Fram and Davis Strait from the early 2000s to mid 2010s^43^. At Fram Strait the Arctic freshwater export increased during 2010–2013^44^, although the absence of a change in the salinity of the East Greenland Current at 60°N (Fig. 4d) suggests the Nordic Seas may act as a buffer of interannual changes from Fram Strait. The Arctic freshwater transport in the LC on the Labrador Shelf increased by an average of 10 ± 2 mSv in 2012–2016 compared to the 2005–2010 period^22^, although it remained close to the 70-year long term (1950–2016)^22^. The cumulative total of an additional ~300 km^3^ in 2012–2016 is the equivalent of <5% of the change in total freshwater content. Thus we conclude that an increase in Arctic freshwater transport within the East Greenland Current or LC was not a major source of additional freshwater in 2012–2016.

Short-lived changes in seasonal wind fields can force fresh water from the boundary currents into the interior of the Labrador and Irminger Sea causing surface salinity anomalies on interannual scales^45–47^. However, our results show no evidence of this process being enhanced throughout the 2012–2016 period, because the interior Irminger and Labrador Seas upper 200 m remained close to mean conditions throughout (Figs. 2 and 3). Further, while the EGC can receive additional freshwater from enhanced Greenland ice-sheet melt, the volume of freshwater from ice sheet melt is small compared to Arctic freshwater sources, and cannot explain interannual variations in SPNA salinity^48^.

The entire SPNA region accumulated an extra 6600 km^3^ of freshwater in 2012–2016. Of that total, 5900 km^3^ accumulated in the eastern basins (Iceland Basin and Rockall Trough). In all, 95% of the freshwater content anomaly in the eastern basins can be accounted for by the three mechanisms above. The re-routing of LC-Arctic water supplied +4600 km^3^, the net precipitation anomaly provided +700  km^3^, and a small increase in Arctic freshwater export provided+300 km^3^.

**Fig. 8:**
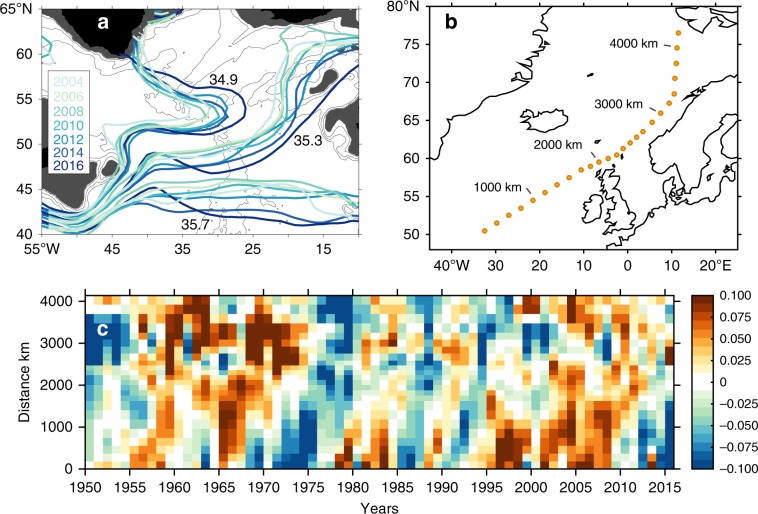
Changing distribution of surface salinity. **a** Annual mean locations of the 34.9, 35.3 and 35.7 isohalines in the upper 500 m layer, from 2004 to 2016 (from EN4, see colour key inset); **b** Station locations for salinity anomalies shown in lower panel; **c** Time-distance plot of 0–200 m annual salinity anomalies (mean period 1950–2016) along the approximate advection pathway of the North Atlantic Current, illustrating the 4–6 year propagation time for positive and negative anomalies from the subpolar North Atlantic and through the Nordic Seas (EN4).

A final potential contributing process that we consider is the expansion of the subpolar gyre. The NAC and subpolar front in the Newfoundland Basin shift meridionally in response to NAO forcing (northward in NAO-positive winters, leading to positive salinity and temperature anomalies in the area south of the mean position of the front^35,49^). This response is associated with a change in the zonal spread of the opposing cold/fresh and warm/saline water masses, and zonal shifts in the location of the boundary between them (the subpolar front) within the eastern NAC complex^10,11^. This change has been interpreted as a gyre that expands and contracts on multi-year timescales in response to buoyancy and wind forcing, and an expanded gyre is characterised by a more eastward spread of cold and fresh water in the upper and intermediate layers, and sometimes with a spin-up of the gyre^11,50^. We find that in the eastern basins, the properties of the upper waters in 2014–2016 (as defined by their potential temperature-salinity relationship) have changed dramatically, indicating that the increase in freshwater content is not simply a vertical migration of isohalines, but a change in water type (Fig. 5f). By 2016 the salinity distribution of the SPNA was radically different to that observed in 2004, with isohalines having shifted up to 700 km to the east and 500 km to the south (Fig. 8a). This implies a large eastward shift of the subpolar front that explains the change in water types found in the eastern basins. We consider how these observations relate to the gyre circulation in the next section and the Discussion.

### Circulation change in response to wind stress curl anomalies

In the previous section we showed that the accelerated freshening observed in the Iceland Basin and Rockall Trough originated in changes in circulation of the Labrador Current. We now consider the forcing for those changes and their relationship with the circulation within the NAC. Changes in wind stress curl associated with winter NAO index anomalies can force a rapid response in ocean circulation, including driving anomalies in the gyres^51^. Here we examine how the SPNA ocean circulation responded to changes in wind stress curl from 2010 to 2016. We show that the change in atmospheric circulation had two notable effects; it forced unusually large amounts of LC-Arctic water off the shelf, and it significantly altered aspects of the circulation of the NAC in 2014–2016.

**Fig. 9:**
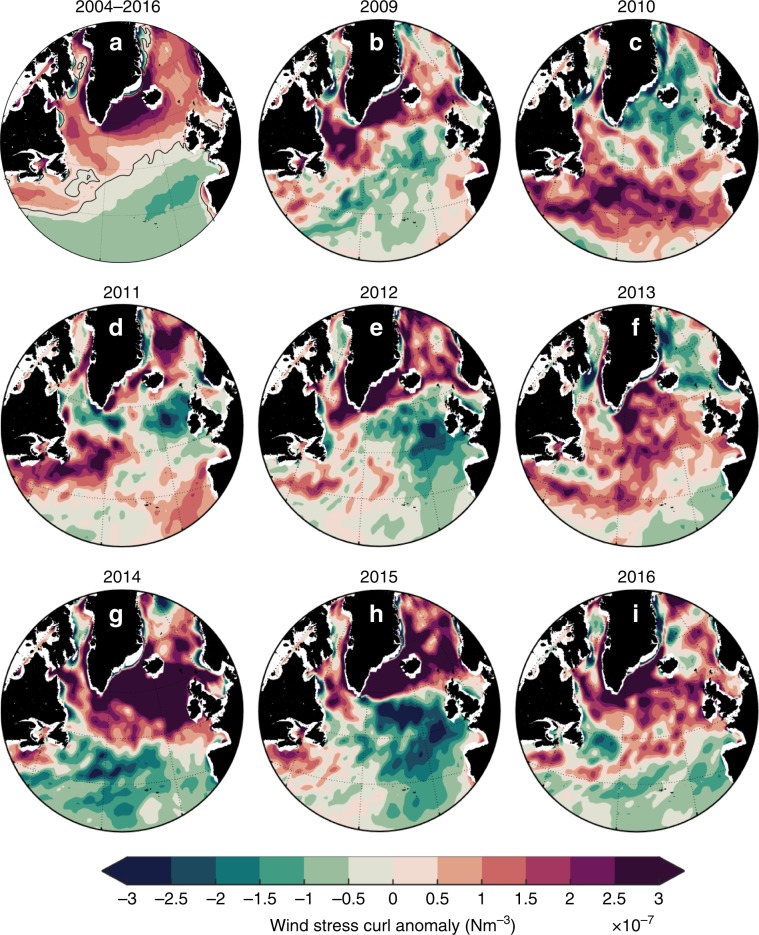
Changes in winter wind stress curl. **a** 2005–2016 mean winter (DJFM) wind stress curl computed from the ERA-Interim reanalysis. **b**–**i** Annual anomalies of winter wind stress curl reference to the 2009–2016 mean. The same colour scale is used for all panels.

In the 2007–2009 period the winter NAO index alternated between positive and negative values, and the wind stress curl pattern was close to the long term mean, with the zero curl line running from the Newfoundland Basin northeastward to Scotland, approximately mapping the main pathway of the NAC (Fig. 9). In the winter of 2010, and to some extent the following winter, there was a sharp drop to a strongly negative winter NAO index and the wind stress curl developed a negative anomaly over the subpolar region and a positive anomaly over the subtropics. This pattern contributed to the observed reduction in the strength of the MOC at 26°N since then^27^. The change in wind stress curl induced a cyclonic circulation anomaly in the region of the Mann eddy (Figs. 1 and 9), and a southward shift of the subpolar front in the Newfoundland Basin, leading to a fresh anomaly in the shallow and 200–1000 m layers there in 2011 (Figs. 2 and 3).

**Fig. 10:**
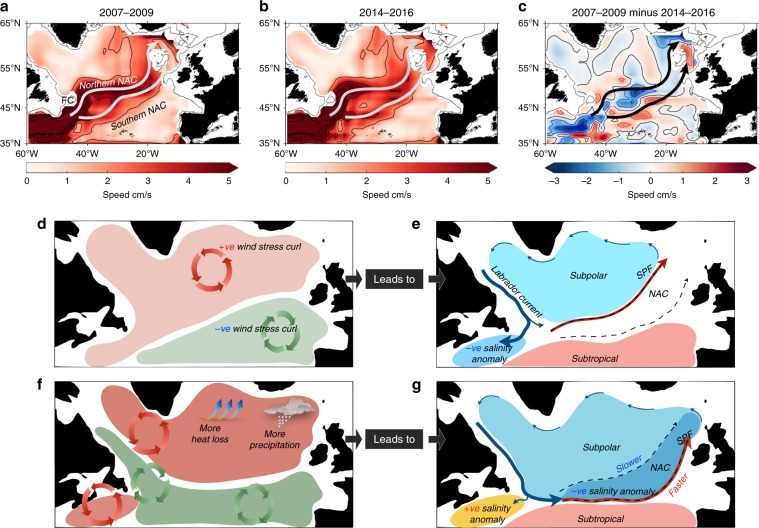
Changed ocean circulation in response to atmospheric forcing. **a**–**c** Geostrophic current speed at 200 m during **a** mean of 2007–2009; **b** mean of 2014–2016; **c** difference between the two periods [(2014–2016)–(2007–2009)]. Speed calculated from geostrophic velocity at 200 m computed from EN4 data, with zero reference velocity at 1200 m. **d**–**g** schematic summary of atmospheric and oceanic processes that impact the salinity and freshwater content of the subpolar region; **d** the mean pattern of windstress curl in 2005–2009 with positive curl over the subpolar region and negative to the southeast; **e** the resulting mean ocean circulation with a cyclonic subpolar gyre, the North Atlantic Current (NAC) zone following the zone of zero and low windstress curl, the Subpolar Front (SPF) located in the northern NAC zone, and the Labrador Current follow a primary pathway along the edge of the continental shelf; **f** wind stress curl pattern in 2014–2016, with stronger positive curl over the subpolar North Atlantic associated with increased heat loss and increased net precipitation, and a zone of negative curl over the Labrador and Newfoundland shelf and Newfoundland Basin; **g** the resulting ocean circulation in 2014–2016 with the Labrador Current following a new primary pathway into the NAC zone, the SPF shifted to the south, a slower northern NAC zone and faster southern NAC zone, and the resulting positive salinity anomaly in the North West Atlantic Continental Shelf and Slope region and negative salinity anomaly in the eastern Subpolar region.

In 2009–2011 the wind stress curl anomaly also shows a band of positive values along the Labrador and Newfoundland shelves and over the Newfoundland Basin (Fig. 9), and that feature is associated with fresh salinity anomalies on the NWACSS, as the LC carries Arctic water southwards along the shelf and slope. In 2012 the winter NAO index switched to strong positive values and the wind stress curl field formed a typical NAO-positive pattern, with a band of negative anomaly along the Labrador and Newfoundland shelves. Negative curl also developed over the open ocean to the east of Newfoundland. This curl pattern is associated with the positive salinity anomalies on the NWACSS (Fig. 2). The anomaly represents a north-eastwards shift of the line of zero curl associated with stronger eastward zonal wind stress and strong Ekman transport off the Newfoundland shelf^51^ (Fig. 10). The ocean response was increased forcing of LC-Arctic water off the shelf and into the NAC. This is consistent with a model study which shows the LC-Arctic transport to be strongly influenced by winter winds^39^.

The winter NAO index was strongly positive in 2014, and a strong positive East Atlantic Pattern was also established: an extreme wind stress curl pattern was set up across the SPNA, with a region of positive anomaly extending as far south as 45°N (Fig. 9). This pattern had a profound effect on the circulation of the NAC and consequently on the geographical reach of the LC water by altering the velocity in the main NAC branches. The speed of the northern NAC branch reduced in 2014–2016 (Fig. 10, Supplementary Fig. 1, Supplementary Note 1), a process which in itself can induce a decrease in salinity at a fixed point if there is a negative salinity gradient along the current pathway^52^. In contrast, the speed of the southernmost branch increased and its pathway extended further east (Fig. 10 and Supplementary Fig. 1). This increase in speed is associated with the southward and eastward relocation of the subpolar front (Fig. 8), and thus represents a shift of the baroclinic front away from the northern branch and into the southern branch.

Finally we note that during this period, anomalously strong surface winter heat loss from the ocean to the atmosphere, and the subsequent deep winter mixing contributed to the development of an extreme cold anomaly north of the NAC in the central SPNA^53,54^. The production of large volumes of subarctic mode waters can force a dynamic gyre response in the form of zonal movement of fronts^10^ and this process has likely contributed to the changes we observe in the baroclinic velocity of the NAC, and hence to the salinity anomalies.

In summary, the response of the LC and NAC circulation to the change in wind stress curl associated with positive NAO and East Atlantic Pattern conditions had three important consequences in 2012–2016 that accelerated a period of freshening that began after 2008. First, starting in 2012 the shallow LC-Arctic water was re-routed off the Newfoundland shelf and into the NW Corner and an unusually large amount of freshwater spread into the NAC, initiating a rapid increase in freshwater content of the SPNA. The NWACSS area simultaneously experienced a rapid increase in salinity as it was deprived of LC-Arctic water. Second, in 2014–2016 the main NAC branch that feeds the west Iceland Basin slowed because the subpolar front shifted to the location of southern branch of the NAC, increasing its speed and extending it unusually far to the east (i.e. an expansion of the gyre). The southern branch was atypically fresh because it was carrying the salinity anomaly caused by the unusual addition of fresh water masses (including LC-Arctic water, Subarctic Intermediate water, and Labrador Sea Water) from the Newfoundland basin. Third, an increase in precipitation associated with the unusual atmospheric circulation acted to reinforce the freshening caused by changes in circulation. Thus the extremely fresh 2014–2016 eastern basin conditions reflect a combination of processes, mostly involving changes in ocean circulation, that were in turn linked to the particular sequence of large changes in atmospheric circulation that occurred over the North Atlantic during 2012–2016.

## Discussion

We have shown that in 2012–2016 the subpolar North Atlantic underwent a basin-scale freshening that is more rapid and with a larger magnitude than any changes observed in the previous five decades. Additionally, the salinity in the eastern basins reached a level lower than any records have shown for the past 120 years. This massive and rapid increase in freshwater content of the region resulted primarily from large scale changes in ocean circulation driven by atmospheric forcing.

Much has been written about the causes of the Great Salinity Anomaly in the late 1960s and early 1970s, but 2012–2016 event does not share the same characteristics. Most notable is that the 2012–2016 event was not evident in the Labrador Sea: during the Great Salinity Anomaly there was enhanced freshwater export through the Fram Strait, and the wind pattern (negative NAO and negative East Atlantic Pattern) forced additional freshwater of the Greenland shelves to spread directly over the Labrador Sea. Thus the processes driving the 2012–2016 event are not the same as those of the Great Salinity Anomaly 50 years earlier.

We have shown that anomalously strong wind stress curl in winters 2012–2016 increased the freshwater convergence in the subpolar region by re-routing the LC-Arctic eastward off the Newfoundland shelf, by shifting the baroclinic subpolar front to the southern branch of the NAC, and by extending southern branch further to the east. The identified linkage between the wind stress curl pattern and changes in the NAC characteristics is consistent with earlier studies suggesting that a stronger cyclonic wind stress curl over the SPNA leads to cooler fresher conditions. We have no direct evidence to say whether this is related to a reduced penetration of warm, saline subtropical water into the region as previously argued^11^, but we conclude that a new mechanism (redistribution of Arctic water) is important in generating the extreme salinity anomalies observed in 2012–2016. Studies have shown that a weak MOC favours the inclusion of subarctic water into the NAC east of the Grand Banks and this process has been linked with an intensification of the subpolar gyre circulation^10,55^ and an SPNA interior cooling and freshening^56,57^. Here we add nuance to the picture; during the 2012–2016 period, the increased speed of the gyre is restricted to the southern branch of the NAC, and not the northern branch, which slowed when the baroclinic front moved zonally. Our results illustrate that these dynamical changes, initiated by the wind stress curl and buoyancy forcing, are directly reflected in the freshwater pathways and hence, larger-scale freshwater variability in the subpolar region. Consequently, the exceptionally strong atmospheric forcing, reported for the winters 2014–2016 and which caused significant heat loss^54^ also contributed to an exceptional freshwater redistribution in the North Atlantic.

Our results provide some clarity around a recent debate, in which the concept of a subpolar gyre expanding and contracting zonally (and possibly associated with a spin-up or slowdown) in response to atmospheric forcing was recently called into question^55,58^. At the heart of the debate lies a lack of clarity in the definition of the gyre and the subpolar front and its relationship with the branches of the NAC. The front is a boundary between the cold/fresh Arctic/subpolar water masses, and the warm/saline subtropics-dominated water masses, and is dynamically connected to the baroclinic current cores of the NAC due to the geostrophic equation. We have shown that the subpolar front can shift location; meridionally in the Newfoundland Basin, and zonally in the eastern basins. The location is dependent on where (and how much) LC-Arctic water mixes with and modifies the originally warm/saline water in the NAC^59^. It is also dependent on the zonal reach of the southern branch (the latter does not form a closed streamline and was therefore not considered part of the gyre by ref. ^55^). Defining the extent of the gyre by the location of the spread of cold/fresh water (i.e. location of the subpolar front and its baroclinic current) gives a view of the gyre expanding and contracting. In contrast, defining the gyre by the location of the northern NAC branch alone (which forms a closed, mainly barotropic streamline) results in a view of the gyre that does not change shape or size. Our results confirm that both interpretations are consistent with the observations, but the expanding subpolar gyre is more clearly described as the expanding spread of subpolar water masses, a zonal shift of salinity and density isolines, and a zonal shift of the baroclinic NAC current. The zonal shift of the subpolar front and the subsequent impact on the speed of NAC branches may be confounding attempts to find a link between the size and strength of circulation of the gyre itself.

The investigated freshwater event was the largest for nearly five decades across the SPNA as a whole, and for 120 years in the Iceland Basin, revealing a high sensitivity of the subpolar gyre dynamics and large-scale hydrographic characteristics to interannual changes in the atmospheric circulation patterns. Potential future changes in the atmospheric forcing, such as the NAO^60^ and associated wind stress curl patterns will have direct consequences for the basin-wide SPNA freshwater content and the properties received downstream, and for the distribution of hydrographic properties within the SPNA and on the NWACSS.

The diversion of oxygen-rich and nutrient-rich Arctic-origin LC water into the NAC has had profound consequences for the NWACSS as well as for the eastern basins (Iceland Basin and Rockall Trough). Since 2012, the NWACSS has seen a rapid increase in marine heatwaves as well as a reduction in oxygen, associated with the flooding of Gulf Stream water onto the shelf, and serious detrimental impacts on local ecosystems^41,61^. In stark contrast, the ecosystems of the eastern basins have been shown to be stimulated into increased productivity by the arrival of nutrient-rich fresh subpolar water in pulses that were substantially weaker than the event described here^62^. Understanding the ecosystem impact of the 2015 freshening in the Rockall Trough and Iceland Basin will be an important next step.

The freshwater anomaly in the Iceland Basin and Rockall Trough is now propagating into the Irminger and Labrador Seas along the pathway of the subpolar circulation, and into the Nordic Seas^16,63^ (Fig. 2). Figure 8b, c show that historical salinity anomalies have taken 4–6 years to propagate from 50°N, 30°W to Svalbard, so we might expect the Atlantic Waters there to be freshening from 2018 onwards. Changes in salinity and stratification impact the extent of deep convection^3–5^ and contribute to density changes in the overflow waters and the subpolar deep western boundary currents^64^ and hence the MOC^65^. This far-reaching impact of eastern Atlantic salinity anomalies highlights the importance of understanding, and correctly simulating, interactions between the North Atlantic ocean dynamics and the atmosphere circulation for future climate predictions.

## Methods

### Salinity and freshwater

Salinity is reported on practical salinity scale throughout the paper. The subpolar North Atlantic (SPNA) is defined as the region 47–65°N, 0–60°W.

The annual mean ocean freshwater content anomaly (m) has been derived using the EN4 hydrographic data set following the formulation of ref. ^66^ (Eq. 1):

1 $${\mathrm{FWC}} = \int _{z_1}^{z_2} \frac{{\rho (T,S,p)}}{{\rho (T,0,p)}}\frac{{S - S_{{\mathrm{ref}}}}}{{S_{{\mathrm{ref}}}}}{\mathrm{d}}z$$

where *ρ* is density of sea-water is derived based on EN4 temperature (*T*), Salinity (*S*) and depth (*p*). *S*_ref_ is the reference salinity set to 35.0. The depth-integration is between *z*_1_ and *z*_2_, which are defined as 0–1000, 0–200 or 200–1000 m. It should also be mentioned that only grid points with data deeper than 1000 m have been taken into account in the FWC calculation. Note, the FWC anomaly are with respect to the 2005–2016 climatology.

The net freshwater gain maps and time series are produced by adding evaporation and total precipitation based on monthly means of daily forecast accumulation of the first 12 h from ERA interim reanalysis^67^. Consistent with earlier analysis, the annual mean net freshwater gain anomaly maps are calculated with respect to the 2005–2016 climatology. Time series of net precipitation are ERA5 monthly anomalies for 45–65°N, 30–10°W. The ERA5 data was obtained from ECMWF (https://www.ecmwf.int/en/forecasts/datasets/reanalysis-datasets/era5).

The empirical orthogonal function analysis of the salinity fields was carried out following ref. ^68^. Statistical significance of the correlation analysis followed ref. ^69^.

### Wind stress curl

The wind stress curl (Nm^−3^) was calculated as follows (Eq. 2):

2 $$\nabla \times \tau = \frac{{\partial \tau _y}}{{\partial x}} - \frac{{\partial \tau _x}}{{\partial y}}$$

where *τ*_*x*_ and *τ*_*y*_ are the zonal and meridonal wind stress, respectively, based on the monthly means of daily means from the ERA-interim reanalysis^70^. The 2004–2016 climatology was removed from the monthly data before calculating the December-Mars winter averages. DJFM wind-stress curl anomalies are generally considered to best represent the atmospheric circulation. The grid resolution of the data set is 0.75° × 0.75°.

### Velocity

The geostrophic velocities in Fig. 1a were derived from data obtained through the Copernicus Marine Environment Monitoring Service (CMEMS, http://marine.copernicus.eu), and are from CMEMS/DUACS DT2018 (product identification: SEALEVEL_GLO_PHY_L4_REP_OBSERVATIONS_008_047, ref. ^71^). The 1/4° absolute zonal (u_geo_) and meridional (*v*_geo_) surface geostrophic velocities (ms^−1^) are derived based on the 1993–2016 sea surface height, ζ, measurements from the global multimission altimeter satellites following the geostrophic approximation (Eqs. 3 and 4):

3 $$u_{geo} = - \frac{g}{f}\frac{{\partial \zeta }}{{\partial y}}$$

4 $$v_{geo} = \frac{g}{f}\frac{{\partial \zeta }}{{\partial x}}$$

where the gravitational acceleration is, *g*, and the Coriolis parameter is, *f*.

The currents at 200 m shown in Fig. 10 are computed from EN4 data: geostrophic velocity at 200 m referenced to zero velocity at 1200 m. Currents from from the observation-based product ARMOR3D^72,73^ are shown in Supplementary Fig. 1 for comparison to Fig. 10. The ARMOR3D product merges different sources of observations (altimetry and T and S profiles) in order to estimate weekly global temperature, salinity, geopotential height and geostrophic current from the surface down to 1500 m. In a first step the steric part of sea level anomaly is projected along the water column through statistical profiles to estimate gridded T/S fields. This step brings mesoscale patterns close to altimetry. In a second step in-situ T/S profiles are merged to correct the previous estimate. Finally, geostrophic currents are estimated through the thermal wind equation referenced at the surface with altimetry. In Fig. 10 ARMOR3D current are filtered at 3.5° in latitude and 4.5° in longitude in order to remove high energetic smaller scales.

### Reporting summary

Further information on research design is available in the Nature Research Reporting Summary linked to this article.

## Supplementary information

Supplementary Information

Reporting Summary

## Data Availability

All data used in this analysis are available as follows. The EN4 data set (ref. ^74^ version 4.2.0) with the bias correction of ref. ^75^ was obtained from https://www.metoffice.gov.uk/hadobs/en4/, downloaded on the 1 November, 2017. The time series of salinity of the upper ocean (Faroe-Shetland Channel, Rockall Trough, Faroe Bank Channel, Iceland Basin, shown in Fig. 4a) were obtained from ICES (https://ocean.ices.dk/iroc/, downloaded November 2018). The surface salinity data (Fig. 4c) are made freely available by the French Sea Surface Salinity Observation Service (http://www.legos.obs-mip.fr/observations/sss/) (downloaded November 2018), 10.6096/SSS-BIN-NASG. Time series of annual salinity anomalies of the upper ocean (0–500 m) in the Labrador Sea (Fig. 4b) are available on request from Igor.Yashayaev@dfo-mpo.gc.ca. The anomalies are relative to a mean seasonal cycle computed at a high-resolution topographically-adjusted spatial grid, and averaged over the whole Labrador Sea. Salinity data at OSNAP mooring M4 in the Iceland Basin (58.0°N, 21.1°W, Fig. 4e) are available at 10.7924/r42n52w51. The Extended Ellett Line programme consists of repeat hydrographic sections along a line from Iceland to Scotland^12^ (Figs. 1b and 5); data are available from https://www.bodc.ac.uk. The OVIDE^8^ programme consists of repeat hydrographic sections along a line from Greenland to Portugal (Figs. 1b and 5). Data from the 2014 section are available from 10.17882/52153 (https://www.seanoe.org/data/00410/52153/) and OVIDE-BOCATS 2016 on request from pascale.lherminier@ifremer.fr, see https://archimer.ifremer.fr/doc/00480/59190/61877.pdf. This analysis used E.U. Copernicus Marine Service Information: ARMOR3D fields available through MULTIOBS_GLO_PHY_REP_015_002 product and DUACS DT2018 through SEALEVEL_GLO_PHY_L4_REP_OBSERVATIONS_008_047 product.

## Code availability

The computer codes used to analyse the data are available from the corresponding author on reasonable request.
