# Supplementary material for: Ocean circulation causes the largest freshening event for 120 years in eastern subpolar North Atlantic
Source: Nat Commun. 2020 Jan 29;11:585. doi: 10.1038/s41467-020-14474-y (PMC6989661; doi:10.1038/s41467-020-14474-y)
Supplement: Supplementary file 1 — Supplementary Information [file 41467_2020_14474_MOESM1_ESM.docx]

Supplementary Information to Holliday et al., Ocean Circulation Causes the Largest Freshening Event for 120 Years in Eastern Subpolar North Atlantic. *Nature Communications*.

**Supplementary Information**

**Ocean Circulation Causes the Largest Freshening Event for 120 Years in Eastern Subpolar North Atlantic**

N. Penny Holliday^1^*, Manfred Bersch^2^, Barbara Berx^3^, L**é**on Chafik^4^,

Stuart Cunningham^5^ Cristian Florindo-López^1^ Hjálmar Hátún^6^, William Johns^7,^

Simon A. Josey^1^, Karin Margretha H. Larsen^6^, Sandrine Mulet^8^, Marilena Oltmanns^9^,

Gilles Reverdin^10^, Tom Rossby^11^, Virginie Thierry^12^, Hedinn Valdimarsson^13^,

Igor Yashayaev^14^

** corresponding author: penny.holliday@noc.ac.uk*

*1 National Oceanography Centre, Southampton, UK*

*2 Institute of Oceanography, University of Hamburg, Germany*

*3 Marine Scotland Science, Aberdeen, Scotland, UK*

*4 Department of Meteorology and Bolin Centre for Climate Research, Stockholm University, Stockholm, Sweden*

*5 Scottish Association for Marine Science, Oban, Scotland, UK*

*6 Faroe Marine Research Institute, Tórshavn, Faroe Islands.*

*7 Department of Ocean Sciences, Rosenstiel School of Marine and Atmospheric Science, University of Miami*

*8 CLS, France*

*9 Ocean Circulation and Climate Dynamics, GEOMAR Helmholtz Centre for Ocean Research Kiel, Kiel, Germany*

*10 Sorbonne Université, CNRS/IRD/MNHN (LOCEAN), Paris, France*

*11 Graduate School of Oceanography, University of Rhode Island, Kingston, USA*

*12 Ifremer, Univ. Brest, CNRS, IRD, Laboratoire d’Océanographie Physique et Spatiale, IUEM, France*

*13 Marine and Freshwater Research Institute, Reykjavik, Iceland*

*14 Bedford Institute of Oceanography, Dartmouth, Nova Scotia, Canada*

**Supplementary Figures**

Supplementary Figure 1. Intensity of the total (barotropic and baroclinic) current over (a) 2007-2009 and (b) 2014-2016 at 200 m from ARMOR3D field filtered with a LOESS filter at 3.5° in latitude and 4.5° in longitude (see Methods for information about ARMOR3D). (c) Difference of mean current speed between the 2 periods [(2014-2016)-(2007-2009)].

**Supplementary Note 1**

In Figure 10 (main article) we show mean current speeds at 200m during two different periods (2007-2009, and 2014-2016) and the difference between them, in order to highlight the slowing down of the northern NAC (North Atlantic Current) and the increased speed in the southern NAC associated with a shift of the Subpolar Front (SPF) in the second period. The speed is derived from geostrophic velocity referenced to zero at 1200m computed from the EN4 dataset, and thus excludes barotropic velocity. We have few direct measurements of total or absolute velocity in the open ocean, and none in the central NAC zone which is our region of interest here. However there are ocean products that estimate total velocity, and in Supplementary Figure 1 we show speed from total velocity at 200m from one such product, the ARMOR3D (see Methods for details) to test whether including the barotropic velocity changes the conclusion.

The fields from the two data sources are consistent, and thus our conclusions are robust. Both Fig. 10 and Supplementary Figure 1 show that the mean speed of the northern NAC branch where it crosses the Atlantic approximately zonally, is 4-5 cms^-1^ during the first period (2007-2009), and that it slows by 1-2 cms^-1^ in 2014-2016. The southern NAC has mean speeds of 3-4 cms^-1^ in 2007-2009, and is faster by ~1 cms^-1^ in 2014-2016. In Fig. 10 the southern NAC branch has moved further east, resulting in anomalies of 3-4 cms^-1^, in Supplementary Figure 1 the anomalies are smaller (2 cms^-1^) and the eastward displacement of the southern NAC branch is less clear.

The relevance of the magnitude of the change in speed in 2014-2016 (1-2 cms^-1^) can be assessed by considering the potential change in transport of those branches. In Fig. 10 and Supplementary Figure 1, the width of the NAC northern branch, defined as the regions where speeds are greater than 4 cms^-1^, is around 400 km. A decrease in velocity of 1-2 cms^-1^ over 500m depth and 400 km width is the equivalent of -2-4 Sv. The mean total NAC transport has been estimated at 15.5 ±0.8 Sv (Supplementary Reference 1), so a change of 2-4 Sv represents 13-25% of the total NAC transport.

**Supplementary Reference**

1. Sarafanov A., et al., Mean full-depth summer circulation and transports at the northern periphery of the Atlantic Ocean in the 2000s, *J. Geophys. Res*., 117, C01014, doi:10.1029/2011JC007572. (2012)
