# Supplementary material for: Ocean circulation causes the largest freshening event for 120 years in eastern subpolar North Atlantic
Source: Nat Commun. 2020 Jan 29;11:585. doi: 10.1038/s41467-020-14474-y (PMC6989661; doi:10.1038/s41467-020-14474-y)
Supplement: Supplementary file 2 — Reporting Summary [file 41467_2020_14474_MOESM2_ESM.pdf]

nature research

Corresponding author(s): NP Holliday

Last updated by author(s): Oct 25, 2019

Reporting Summary

Nature Research wishes to improve the reproducibility of the work that we publish. This form provides structure for consistency and transparency in reporting. For further information on Nature Research policies, see Authors & Referees and the Editorial Policy Checklist.

Statistics

For all statistical analyses, confirm that the following items are present in the figure legend, table legend, main text, or Methods section.

n/a	Confirmed	
☒	☐	The exact sample size (*n*) for each experimental group/condition, given as a discrete number and unit of measurement
☒	☐	A statement on whether measurements were taken from distinct samples or whether the same sample was measured repeatedly
☒	☐	The statistical test(s) used AND whether they are one- or two-sided<br>*Only common tests should be described solely by name; describe more complex techniques in the Methods section.*
☒	☐	A description of all covariates tested
☒	☐	A description of any assumptions or corrections, such as tests of normality and adjustment for multiple comparisons
☒	☐	A full description of the statistical parameters including central tendency (e.g. means) or other basic estimates (e.g. regression coefficient) AND variation (e.g. standard deviation) or associated estimates of uncertainty (e.g. confidence intervals)
☒	☐	For null hypothesis testing, the test statistic (e.g. *F*, *t*, *r*) with confidence intervals, effect sizes, degrees of freedom and *P* value noted<br>*Give P values as exact values whenever suitable.*
☒	☐	For Bayesian analysis, information on the choice of priors and Markov chain Monte Carlo settings
☒	☐	For hierarchical and complex designs, identification of the appropriate level for tests and full reporting of outcomes
☒	☐	Estimates of effect sizes (e.g. Cohen's *d*, Pearson's *r*), indicating how they were calculated

Our web collection on statistics for biologists contains articles on many of the points above.

Software and code

Policy information about availability of computer code

Data collection	no software
Data analysis	Matlab tools

For manuscripts utilizing custom algorithms or software that are central to the research but not yet described in published literature, software must be made available to editors/reviewers. We strongly encourage code deposition in a community repository (e.g. GitHub). See the Nature Research guidelines for submitting code & software for further information.

Data

Policy information about availability of data

All manuscripts must include a data availability statement. This statement should provide the following information, where applicable:

- Accession codes, unique identifiers, or web links for publicly available datasets
- A list of figures that have associated raw data
- A description of any restrictions on data availability

All data used in this analysis are available as follows. The EN4 data set (ref74 version 4.2.0) with the bias correction of ref 75 was obtained from https://www.metoffice.gov.uk/hadobs/en4/, downloaded on the 1st of November, 2017.
The time series of salinity of the upper ocean (Faroe-Shetland Channel, Rockall Trough, Faroe Bank Channel, Iceland Basin, shown in Fig. 4a) were obtained from ICES (https://ocean.ices.dk/iroc/, downloaded November 2018). The surface salinity data (Fig. 4c) are made freely available by the French Sea Surface Salinity Observation Service (http://www.legos.obs-mip.fr/observations/sss/) (downloaded November 2018), https://dx.doi.org/10.6096/SSS-BIN-NASG. Time series of annual salinity anomalies of the upper ocean (0-500m) in the Labrador Sea (Fig. 4b) are available on request from Igor.Yashayaev@dfo-mpo.gc.ca. The anomalies are relative to a mean seasonal cycle computed at a high-resolution topographically-adjusted spatial grid, and averaged over the whole Labrador Sea. Salinity data at OSNAP mooring M4 in the Iceland Basin (58.0°N, 21.1°W, Fig. 4e) are available at https://doi.org/10.7924/r42n52w51.
The Extended Ellett Line programme consists of repeat hydrographic sections along a line from Iceland to Scotland12 (Figs. 1b, 5); data are available from https://www.bodc.ac.uk. The OVIDE8 programme consists of repeat hydrographic sections along a line from Greenland to Portugal (Figs. 1b, 5). Data from the 2014

section are available from doi:10.17882/52153 (https://www.seanoe.org/data/00410/52153/) and OVIDE-BOCATS 2016 on request from pascale.lherminier@ifremer.fr, see https://archimer.ifremer.fr/doc/00480/59190/61877.pdf.
This analysis used E.U. Copernicus Marine Service Information: ARMOR3D fields available through MULTIOBS_GLO_PHY_REP_015_002 product and DUACS DT2018 through SEALEVEL_GLO_PHY_L4_REP_OBSERVATIONS_008_047 product.

Field-specific reporting

Please select the one below that is the best fit for your research. If you are not sure, read the appropriate sections before making your selection.

☐ Life sciences ☐ Behavioural & social sciences ☒ Ecological, evolutionary & environmental sciences

For a reference copy of the document with all sections, see nature.com/documents/nr-reporting-summary-flat.pdf

Ecological, evolutionary & environmental sciences study design

All studies must disclose on these points even when the disclosure is negative.

Study description	analysis of ocean and atmospheric data
Research sample	Data sets of physical properties: temperature, pressure, salinity, net precipitation, wind
Sampling strategy	all available data were used
Data collection	no new data collection
Timing and spatial scale	Data from 1950 to 2016, over the North Atlantic ocean between 30-65N
Data exclusions	none
Reproducibility	We make use of available data and explain what we do - anyone could reproduce our results from the same data.
Randomization	n/a
Blinding	n/a

Did the study involve field work? ☐ Yes ☒ No

Reporting for specific materials, systems and methods

We require information from authors about some types of materials, experimental systems and methods used in many studies. Here, indicate whether each material, system or method listed is relevant to your study. If you are not sure if a list item applies to your research, read the appropriate section before selecting a response.

Materials & experimental systems

n/a	Involved in the study	
☒	☐	Antibodies
☒	☐	Eukaryotic cell lines
☒	☐	Palaeontology
☒	☐	Animals and other organisms
☒	☐	Human research participants
☒	☐	Clinical data

Methods

n/a	Involved in the study	
☒	☐	ChIP-seq
☒	☐	Flow cytometry
☒	☐	MRI-based neuroimaging
